# Pedagogical introductions to sex and gender diversity in anatomy: Exploring student perspectives

**DOI:** 10.1002/ase.70330

**Published:** 2026-08-25

**Authors:** Charlie W. Brake, Charys M. Martin

**Affiliations:** ^1^ Schulich School of Medicine & Dentistry, Department of Anatomy and Cell Biology Western University London Ontario Canada

**Keywords:** anatomy education, gender and sex diversity, pedagogical interventions, student perspectives

## Abstract

Anatomical education has historically relied on binary terminology, reinforcing the conflation of sex, gender, and gender identity through long‐standing social and linguistic norms. To better include gender and sex diverse students and to respect the delineations between gender and sex, educators are incorporating diversity into their courses through educational primers; however, there is a lack of literature on the subject. Thus, this study explores learners' perceptions and attitudes about adding sex and gender diversity into anatomical education programs at a Canadian university. A total of 73 participants responded to a survey informed by grounded theory, and descriptive statistics are used for analysis. Nearly half of learners felt their overall educational experience was enhanced by the inclusion of sex and gender diversity content in their anatomy course and around half of learners felt more knowledgeable about inclusive language. Some learners were inspired to begin using inclusive language following their anatomy course. Overall, these findings suggest that integration of gender and sex diversity could be well‐received and valued by anatomy students for humanistic skill development. Emerging themes requiring qualitative insights, including learners' definitions of “inclusive language” and the viability of peer‐based learning, will be explored in future work.

## INTRODUCTION

Definitions of sex and gender vary across disciplines, but both are increasingly understood as distinct, multidimensional concepts. Sex encompasses a range of physiological and anatomical attributes, such as chromosomes, hormones, gene expression, and genitalia, though these may not align neatly, as seen in intersex variations.^1^, ^2^, ^3^, ^4^, ^5^ Although sex is often assigned as binary at birth, it is best described as spectral and variable over time.^6^, ^7^ Gender, by contrast, is a sociocultural construct shaped by norms and expectations around masculinity, femininity, and social roles; it has no biological basis for binarization.^2^, ^4^, ^5^ Gender identity, or one's perception of their gender and expression, is therefore fluid and spectral, and may not align with the expectations of one's sex assigned at birth or with binary sex categories altogether.^1^, ^8^ However, dominant cultural narratives conflate sex and gender, reinforced by gender essentialism: an assumption of fixed, binary relationships between sex and gender expression.^9^ Historically, such narratives pathologized individuals outside the binary, leading to nonconsensual medical interventions to prioritize cisheteronormativity.^10^ These legacies persist in modern medicine and education, where anatomy is often taught through binary frameworks and with sexed and gendered language. Yet, anatomy, like the human body, does not exist in a vacuum; it is embedded within complex social systems that shape how difference is perceived.^10^ Therefore, anatomical education must move beyond binary assumptions and teach the body in its full biological and social context.

Anatomy education is a foundational component of health sciences training at undergraduate, graduate, and professional levels, to varying degrees of depth and complexity. In anatomical education, the human body is organized by functional or regional systems. Reproductive and genital anatomy are often divided along binary sex lines, with ‘male’ structures (penis, testes, prostate, seminal vesicles, ductus deferens) differing from ‘female’ structures (ovaries, uterine tubes, uterus, vagina, clitoris, labia). While this categorization reflects common anatomical phenotypes, it excludes those with variations in sex characteristics, who represent roughly one in 100 live births.^1^ Even for individuals with traditionally sexed phenotypes, anatomical structures may change due to genetic variation, aging, medical transition, or surgery.^4^, ^7^ These variations challenge the assumption that anatomy is immutable, or that it can be strictly categorized by binary sex. Moreover, as historical conflations between sex, gender, and anatomy continue to shape educational norms, binary frameworks risk excluding and pathologizing diversity.^10^ To support more inclusive and accurate health sciences education, scholars have called for anatomical teaching to move beyond binary language wherever possible, particularly when describing sexed and gendered anatomy.^6^, ^7^, ^10^

Inclusive language in anatomical education emphasizes structure, function, and development without reference to sex or gender, thereby avoiding assumptions about identity.^6^ However, medical students often report discomfort using inclusive language during patient interactions, particularly when discussing pelvic or genital anatomy with transgender or gender‐diverse patients.^11^, ^12^, ^13^ This fear of saying the “wrong thing” can hinder participation in small group discussions with peers and may contribute to students' poor confidence in caring for gender‐diverse patients compared with cisgender patients.^11^, ^13^ Although the amount of LGBTQ+‐specific training increases throughout medical education, confidence remains low particularly around trans, nonbinary, and intersex care.^11^, ^12^ To address this, some authors suggest that holistically integrating gender and sex diversity into the curriculum is preferable to short‐term or elective training^11^, ^12^, ^14^, ^15^ because early exposure could enable learners to more effectively apply these concepts throughout their education.^16^

Anatomy, as one of the first courses in many health programs, offers a timely opportunity to build foundations of diversity. When inclusive language is introduced consistently, beginning with clear distinctions between sex and gender identity, learning environments become safer and more affirming for sex‐ and gender‐diverse students.^7^ Recent work by Longhurst et al. supports this approach, as they found anatomy students desire a more inclusive curriculum.^17^ Despite this, the integration of inclusive language and sex and gender diversity in anatomy education remains limited, and most educators lack access to practical guidance or evaluated tools.^12^, ^13^ A handful of educators have described using primers to introduce concepts like the sex spectrum or alternatives to binary terms before teaching reproductive anatomy.^6^, ^10^ However, there is a striking lack of research assessing the impact of such materials on learners. This gap leaves anatomy educators without evidence‐based strategies for implementing inclusive practices. This study highlights students' perceptions of integrating sex and gender diversity, via an educational primer, into university‐level anatomy education, and assesses whether learners value this type of pedagogical intervention. This initial exploration of learners' reception to diversity content in anatomy is a critical step toward developing inclusive, responsive curricula that align with contemporary understandings of sex and gender.

## METHODS

This study used a survey to explore how anatomy learners perceived the addition of sex and gender diversity content, as well as inclusive language, into anatomical education programs at Western University. This was an exploratory, descriptive study designed to inductively gather insight on students' reception of such pedagogical innovations, to inform educational resource development and future research interventions.

### Sex and gender diversity primer

At the start of the fall and winter terms in the 2023–2024 academic year, a sex and gender primer created by the authors was distributed to all educators who actively taught anatomy in Western University's Faculty of Health Sciences and the Schulich School of Medicine & Dentistry. Drawing upon definitions from the Canadian Institutes for Health Research^2^ and supported by contemporary anatomical education literature,^18^ this primer conceptualized sex and gender as multidimensional constructs and provided examples of inclusive anatomical language, which were subsequently reflected in survey item framing. The primer was provided in both infographic and PowerPoint formats for educators, and author CMM also shared a video walkthrough of the slides for educators to post on their course management sites. Educators were later asked to share if and how they implemented the primer, or their own diversity materials, during their anatomy course. Six educators indicated that they provided the primer and/or video to their students. Three educators introduced it at the start of the course and referenced it throughout the course when appropriate; one introduced it at the start of their pelvic anatomy unit; and two provided the content as an optional module for students. One educator introduced gender and sex diversity using a primer of their own design, though it was similar in content, context, and scope to the materials that were provided by the research team.

### Recruitment and participants

To participate in this study, learners must have been enrolled in a human anatomy course which included sex and gender diversity content in some capacity. Learners were recruited from eight anatomy courses across undergraduate, graduate, and professional programs. Recruitment from each anatomy course was timed to align with instructors' delivery of sex and gender diversity content; for example, at the start of sexual/reproductive anatomy units. In‐person presentations by author CWB and learning management system (LMS) course site announcements were used to inform students about the study. Participation was anonymous, voluntary, and explicitly advertised as unrelated to students' activities in their anatomy course. Volunteer participants could follow a link or a QR code to access the online survey. This study was approved by Western University's Non‐Medical Research Ethics Board (#124162).

### Data collection

Data were anonymously collected at a single time point using a 16‐item survey hosted on Qualtrics XM (https://www.qualtrics.com). This exploratory survey was developed based on constructs identified in previous work on gender and sex diversity perceptions in medical education^11^, ^12^ and aligned with the study's objectives, providing initial evidence of content validity. Survey items were structured following AMEE guidelines for style and clarity.^19^ Additionally, definitions of key concepts, drawn from standards set by the Canadian Institutes for Health Research, were provided within the survey to support consistent interpretation of items, contributing to response process validity. The survey underwent iterative review and refinement by the research team to improve clarity and alignment with study constructs.

The study's letter of information was included on the first page of the survey, and participants were required to consent to proceed to the survey items by clicking “I agree” following a consent statement. Participants were permitted to skip any of the survey items following consent. The project's definitions of sex and gender were provided prior to the survey items for participants to reference in their responses. Three multiple choice questions were asked to clarify participant demographics: level of education; sex and gender identities, with the option to self‐describe; and previous sources for learning about sex and gender diversity. Examples of common learning sources identified in the literature were used, and participants could select all that applied to contextualize their previous exposure to diversity concepts.^12^ Participants were asked to retroactively rate their knowledge of inclusive language before the course and to rate their current knowledge of inclusive language, each on a 5‐point Likert scale, between “1 ‐ not knowledgeable at all” and “5 ‐ extremely knowledgeable.*”* Participants were asked about their use of inclusive language, with options to indicate if they were using it before the course, if they started using it during the course, planned to start, or did not intend to use it. If students did not intend to use inclusive language, then they were directed to an open‐text response to explain why. Additional items asked participants to characterize their instructor's approach to inclusive language (e.g., “my instructor role‐modelled inclusive language consistently,” “used binary terminology after defining the meaning of the terms,” “my instructor did not reinforce these topics after initial introduction,” or “other” with an open‐text response), and whether peers used inclusive language consistently. Another item asked if the course materials (e.g., lectures, labs, readings) consistently displayed inclusive language and diversity, with an open‐response option to elaborate if they disagreed. Finally, participants were asked to rate how the introduction of sex and gender diversity changed their experience in anatomy on a 5‐point Likert scale, between “1 – negatively impacted my experience,” “3 – no impact/no enhancement,” and “5 – positively enhanced my experience.”

### Data analysis

Given the exploratory nature of this study, descriptive statistics were used to summarize patterns in participants' responses. As data were collected at a single time point and included retrospective pre‐ratings, the study was not designed to support paired statistical comparisons. Additionally, the aim was to assess students' perceptions rather than their objective knowledge, further supporting the use of descriptive analysis.

Open‐text responses were analyzed thematically by author CWB to identify patterns in participants' perspectives. Each response underwent descriptive coding, and statements were grouped based on shared concepts to develop themes. Participants were permitted to skip survey items; responses that were blank or contained only demographic information were considered incomplete and excluded from analysis.

## RESULTS

Seventy‐three complete survey responses were collected between January and April 2024. Between 65 and 72 participants answered each question, although some participants skipped certain items. All reported percentages and n‐values are relative to the number of responses per item. The sample was predominantly cisgender women (75%, *n* = 54/72), and no participants explicitly identified as sex‐ or gender‐diverse (See Figure 1). Respondents were mainly undergraduates (60%, *n* = 43/72; see Figure 1). The most common sources for learning about gender and sex diversity prior to this study were interactions with peers (85.7%, *n* = 60/70) and formal education, like workplace training or in‐class sessions (75.7%, *n* = 53/70; see Figure 2).

**FIGURE 1:**
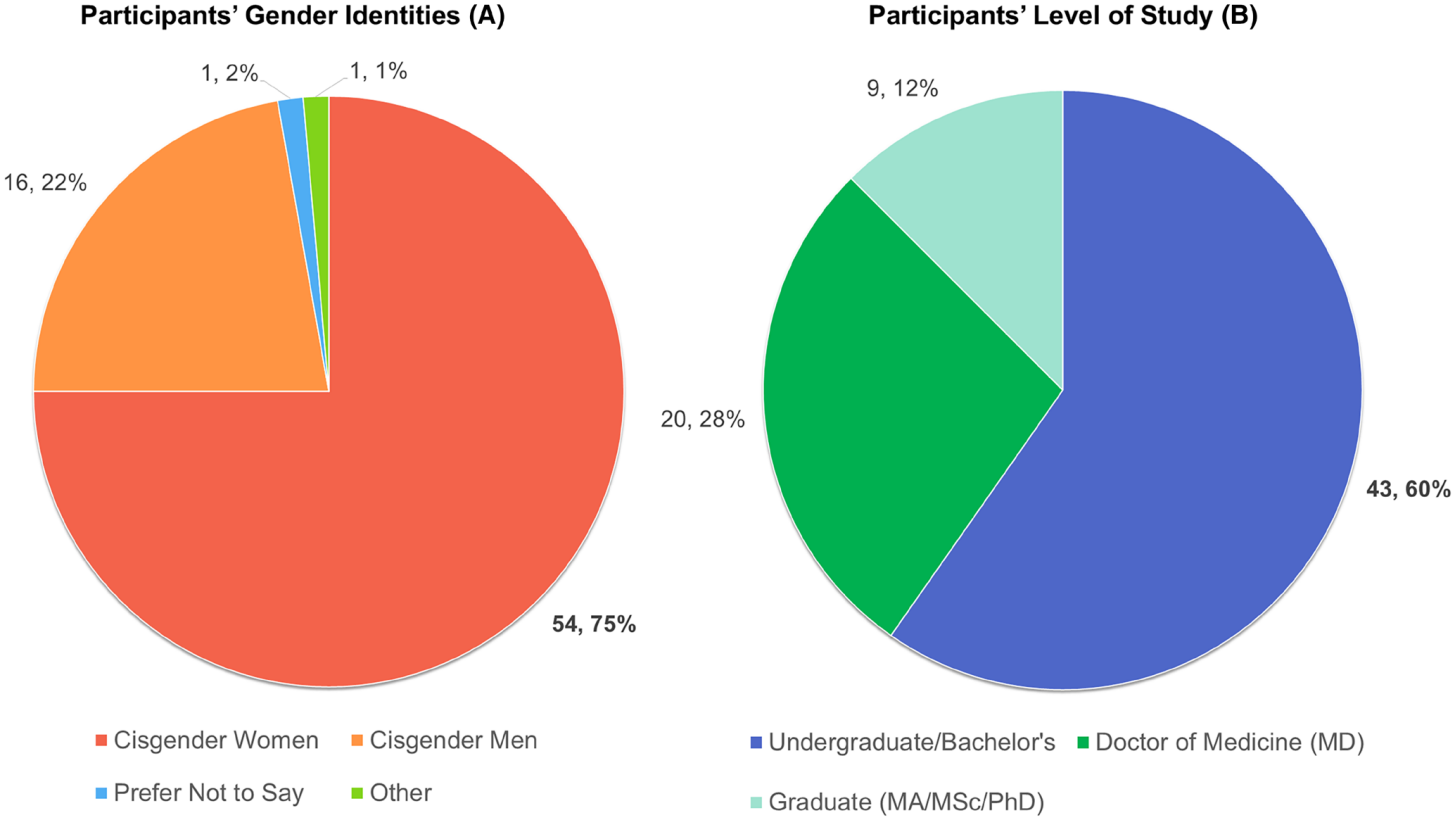
Participants' level of study and gender identities (*n* = 72). Participants' gender identities are pictured in graph A, and their level of study is pictured in graph B. Most participants were undergraduate students who identified as cisgender women. None explicitly identified as sex or gender diverse.

**FIGURE 2:**
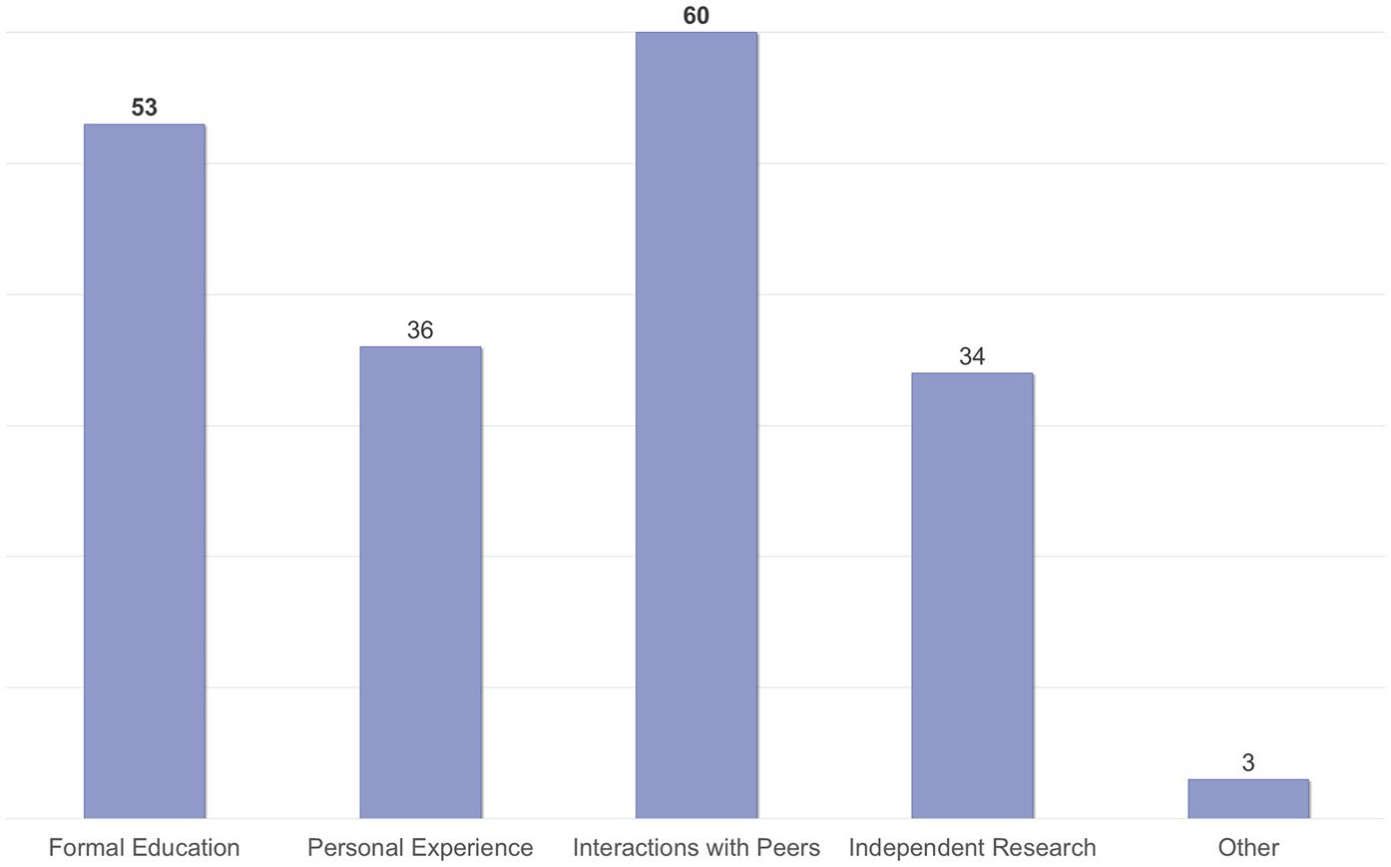
Previous sources of learning about gender and sex diversity (*n* = 70). Respondents were asked to select all that applied. Participants most often learned about diversity prior to their anatomy course(s) through peer interactions (*n* = 60/70) and formal diversity training sessions (*n* = 53/70).

Seventy‐one participants rated their pre‐course knowledge of inclusive language, and 68 rated their post‐course knowledge. Overall, responses shifted toward higher self‐reported knowledge after the course, with more participants identifying as “very knowledgeable” (*n* = 34/68 post‐course vs. *n* = 23/71 pre‐course; see Figure 3). At the same time, a small number of participants rated themselves as less knowledgeable after the course (three vs. two reporting “not knowledgeable at all”), suggesting some variability in perceived knowledge gains.

**FIGURE 3:**
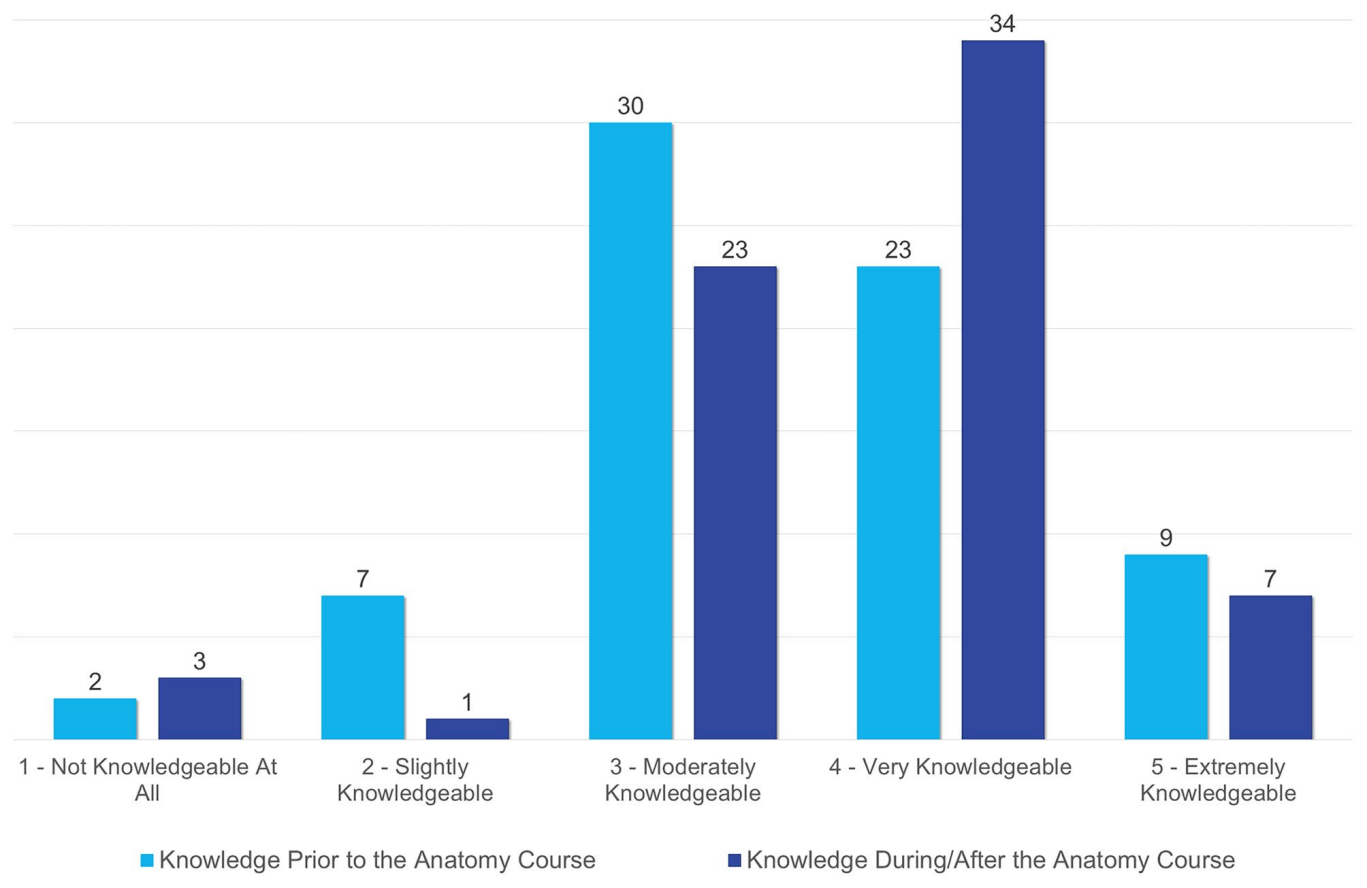
Pre‐course knowledge on using sex and gender inclusive language (*n* = 71) vs. during/post‐course knowledge (*n* = 68). Participants appeared to gain knowledge of inclusive language throughout the course, leading more to identify as ‘very knowledgeable’ at the time of the survey. Some participants may have realized gaps in their understanding on how to use inclusive language, however, leading them to identify as less knowledgeable after exposure to the pedagogical intervention.

Regarding participants' use of, and intentions toward, inclusive language, 69% of respondents stated that they used inclusive language before and during their anatomy course (*n* = 49/71). A combined 17% of participants were inspired to begin using inclusive language either during their anatomy course (6%; *n* = 4/71) or following it (11%, *n* = 8/71). The final 14% indicated that they did not, and would not, use inclusive language (*n* = 10/71; see Figure 4). Six of those participants provided justification. Some stated that inclusive language is misaligned with their beliefs (*n* = 2/6). For example, one participant stated that “sex and gender are interchangeable, and it is binary. Using male/female is inclusive.” Another indicated, “don't feel like I need to. I believe there is only male and female.” Other participants reported that inclusive language is more confusing than binary language (*n* = 2/6); one response read “it's too confusing and then I need to explain myself.” Finally, some believed inclusive language/diversity content is irrelevant to anatomy. One such participant reported, “I try to with peers but when it comes to anatomy and science courses I think we are using sex so there are just scientific facts” (*n* = 2/6; see Figure 5).

**FIGURE 4:**
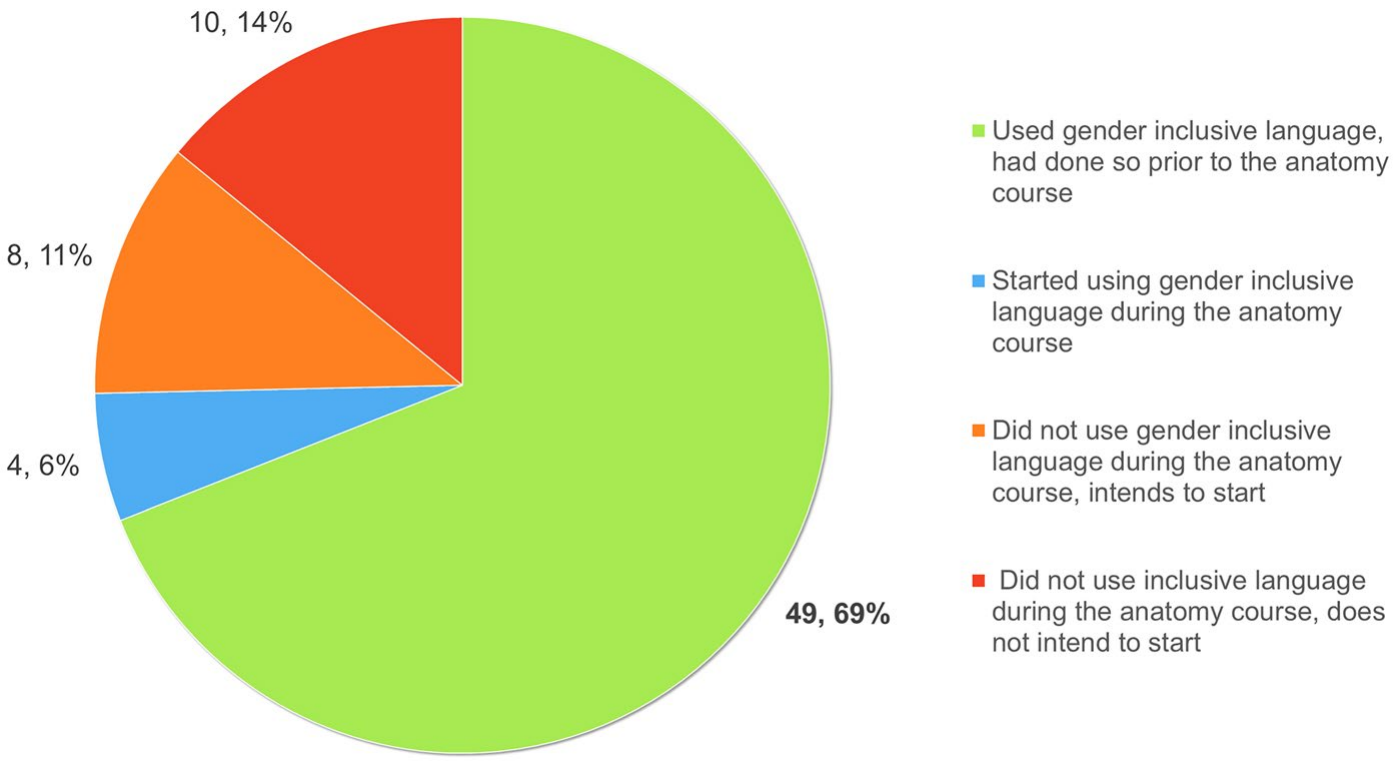
Changes in inclusive language use (*n* = 71). Most participants believed that they used inclusive language before exposure to the pedagogical intervention, though a minority of students do not intend to use inclusive language.

**FIGURE 5:**
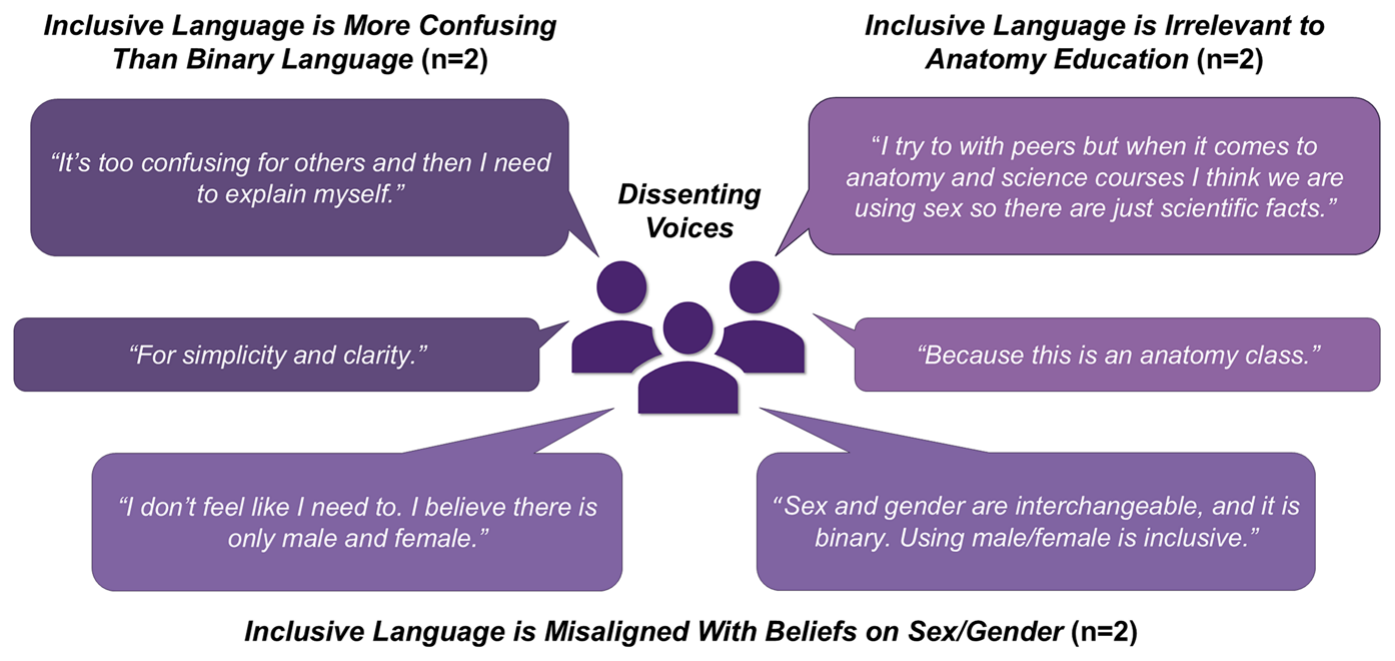
Themes acquired from those who clarified why they do not intend to use inclusive language (*n* = 6). Thematic analysis of the open‐text responses revealed that students who reject inclusive language either disagree with its principles, find it more difficult than using binary language in communications, or believe it to be irrelevant in anatomy education.

Seventy‐two participants reported their instructor's use of inclusive language. Most respondents stated that their instructor modeled inclusive language consistently (62%, *n* = 43/72), but 30% said their instructor still used binary language after providing definitions (*n* = 21/72; see Figure 6). Sixty‐five participants reported on their peers' use of inclusive language, with just over half stating that their peers did not consistently do so (57%, *n* = 37/65; see Figure 6). Relatedly, out of 67 respondents, 78% indicated that course materials were representative of diversity and modeled inclusive language (*n* = 52/67; see Figure 7). Of the 22% who disagreed, five individuals explained why. Open‐text analysis revealed that diversity concepts were minimally included in course materials (*n* = 3/5). Of note, one participant stated that “terms like AFAB/AMAB [assigned female at birth/assigned male at birth] were brought up in one powerpoint, but used male/female after that with infrequent reminders of more inclusive terminology.” Additionally, anatomical education resources did not adequately reflect values of inclusivity (*n* = 2/5). Respondents indicated that “course materials were highly skewed towards AMAB bodies” and that “diagrams and textbooks do not contain terminology that has only become popular in the last 10 years.”

**FIGURE 6:**
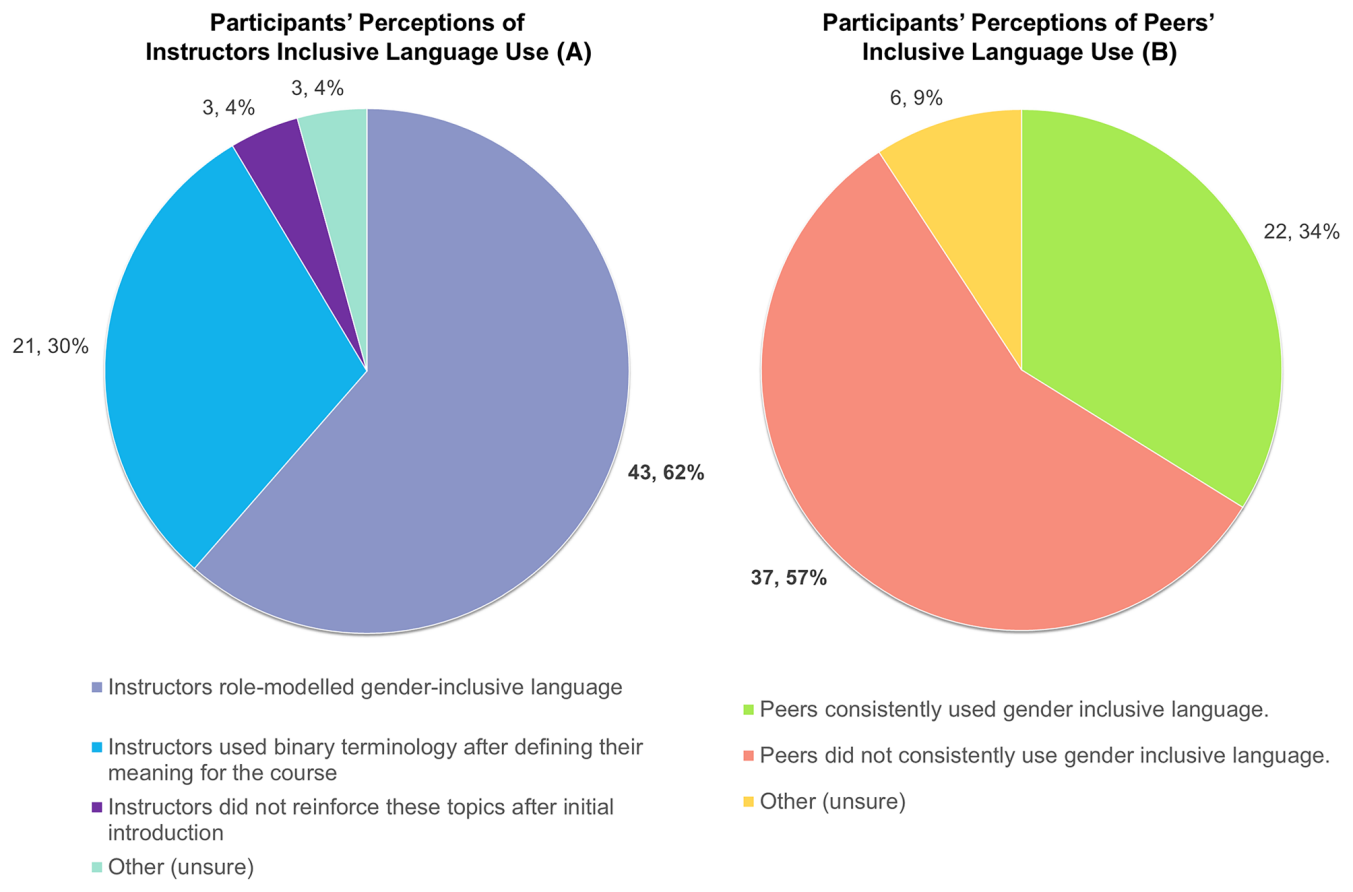
Perceptions of others' inclusive language use. Instructors' inclusive language use is represented in graph A (*n* = 72) and peers' inclusive language use is represented in graph B (*n* = 65). Most participants felt that their instructors modeled inclusive language, but their peers did not model inclusive language.

**FIGURE 7:**
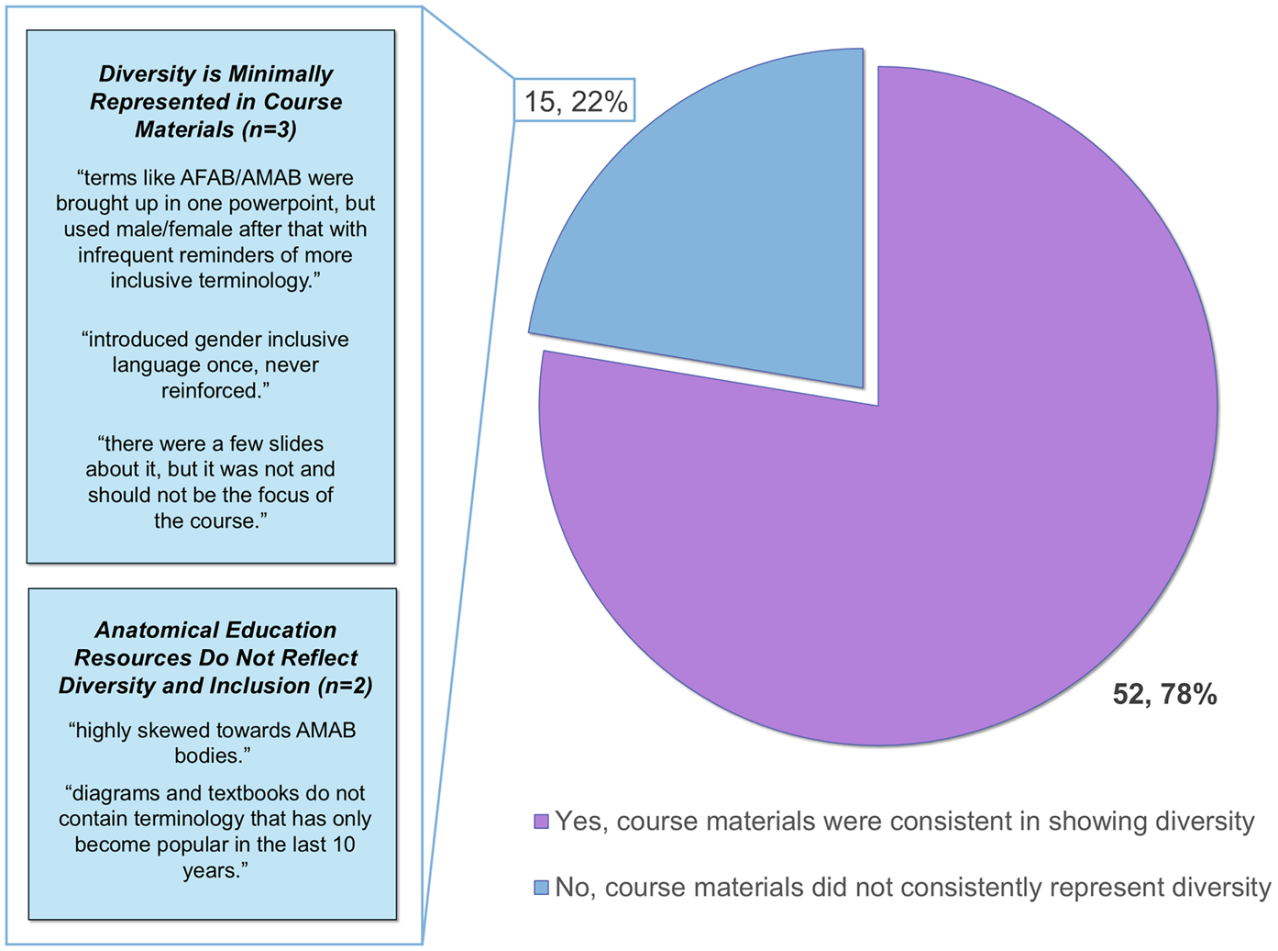
Consistency of sex and gender diverse representation in course materials (*n* = 67). Course materials were mostly perceived as consistently representative, but just under one‐quarter of participants disagreed (*n* = 15/67). The participants who disagreed expressed that professors' course materials did not cover diversity equally throughout the course, and that general anatomical education resources insufficiently reflect diversity.

Sixty‐six participants showed how sex and gender diversity content impacted their experience in anatomical education. A combined 55% of learners indicated that their learning experience was enhanced (*n* = 12/66, 18%) or somewhat enhanced (*n* = 21/66, 32%) and 41% felt neutral (*n* = 27/66). Only a combined 9% felt their experience was somewhat negatively (*n* = 2/66, 3%) or negatively affected (*n* = 4/66, 6%) by the inclusion of diversity content in their anatomy course (see Figure 8).

**FIGURE 8:**
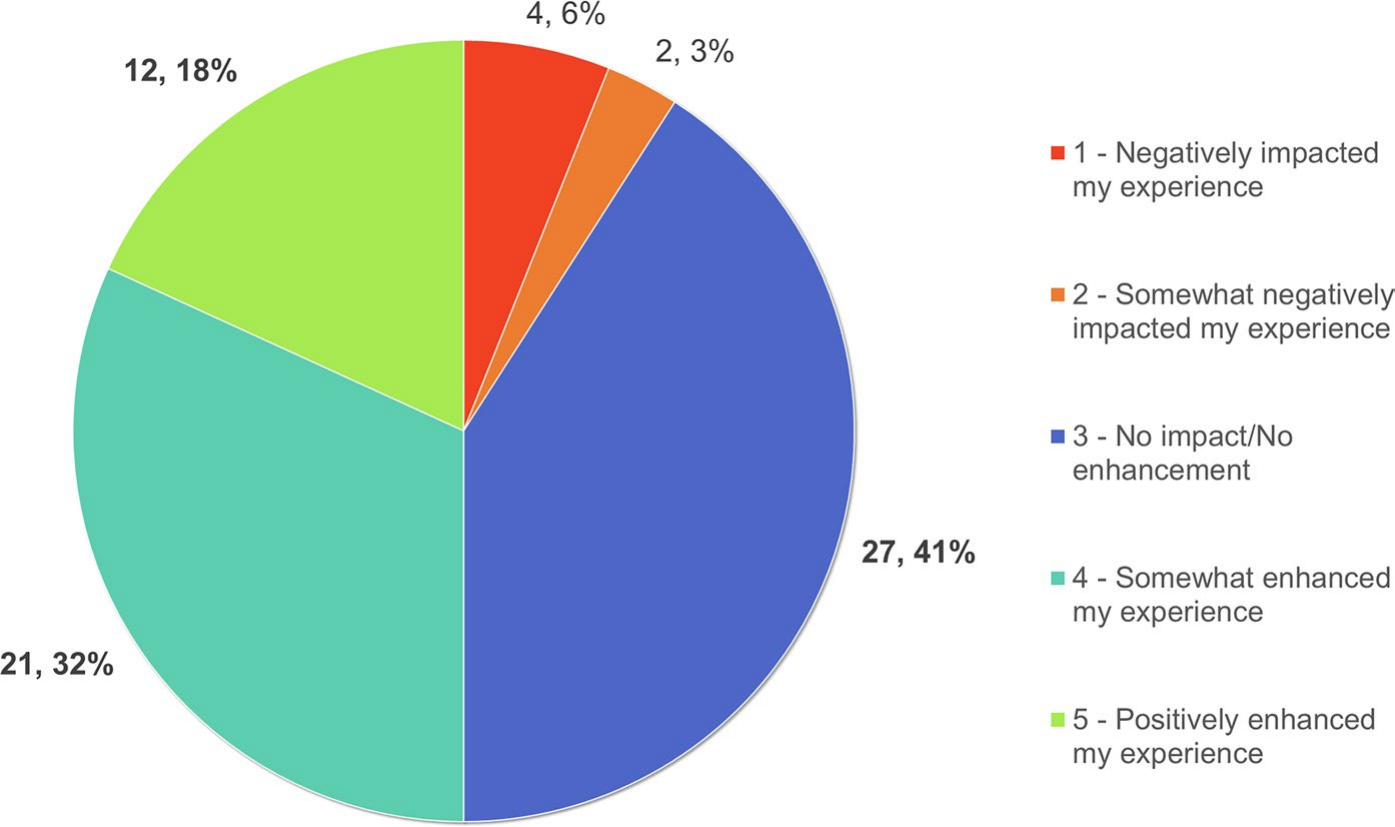
Enhancement of overall learning experience from inclusion of sex and gender diversity in anatomy (*N* = 66). Overwhelmingly, participants either had a positive or neutral experience interacting with diversity content in their anatomy course (combined *n* = 60/66), with minimal negative impact (combined *n* = 6/66). Thus, it is warranted to continue integrating diversity and inclusive language into anatomical education.

## DISCUSSION

To determine students' perceptions of sex and gender diversity in anatomical education, this study surveyed learners after the integration of sex and gender diversity materials within their anatomy courses. Understanding learners' perspectives on this topic is essential to ensure their reception of new diversity materials, which will ultimately lead toward more inclusive anatomical sciences education. Given that most participants felt positively or neutrally toward the inclusion of sex and gender diversity, these results certainly warrant the continued implementation of sex and gender diversity and inclusive language content within anatomical education. These findings are consistent with the literature where Longhurst et al. found that anatomy learners in the UK generally agree that inclusivity is vital to include within anatomical education,^17^ as well as with results of a study by Arthur et al., who reported that medical students desire more curricular opportunities to learn about diverse identities.^11^ Additionally, implementing relatable sex and gender diversity materials could help more students understand the value of this information, seeing as our results indicate that introducing anatomy learners to sex and gender diversity may encourage them to use more inclusive language.

In addition to feeling empowered to use inclusive language or to discuss topics of sex and gender diversity, participants also reported a learning effect from the addition of this content to their anatomy courses. However, a small number of students rated themselves as less knowledgeable, or “not knowledgeable at all,” after their anatomy course. This apparent discrepancy may reflect metacognitive recalibration, where exposure to new or unfamiliar content highlights gaps in understanding. Thus, exposure to diversity content might make some students more aware of what they do not know, reducing overconfidence, as their objective knowledge increased despite their subjective perceptions of knowing less.^20^ In this way, the exposure to inclusive terminology and sex and gender definitions may have fostered deeper self‐awareness, even among those who reported minimal knowledge gains. This phenomenon is consistent with existing literature on inclusive education in health professions, where learners' confidence in their ability to provide gender‐affirming care remains limited despite increased curricular exposure.^11^, ^12^ As such, the observed modest knowledge gains may not reflect a lack of impact, but rather the beginning of a more reflective and accurate self‐assessment process.

When asked to indicate if they used inclusive language during their anatomy course, thematic analysis of dissenting participants' open‐text responses revealed themes of confusion and a belief that sex and gender diversity is irrelevant to anatomy. Confusion surrounding inclusive language might stem from fear of saying the wrong thing, a phenomenon documented in the literature: medical students cite anxiety around inclusive language as a deterrent to participation in small group discussions.^13^ The notion that inclusive language is irrelevant to anatomy, or that sex is scientifically binary, could stem from a lack of diversity within basic biology education. Neither courses nor textbooks adequately represent variations in human sex determination, and often relate sex characteristics with gendered behavior, thus promoting gender essentialism.^9^ Gender essentialism also explains the disconnect between inclusive language use and some participants' beliefs that sex and gender are the same. One participant stated that “sex and gender are interchangeable, and it is binary” and therefore, “using male/female is inclusive.” Thus, a gender‐essentialist definition of ‘inclusive language’ emerged in the data. Because this opposes the formal definition from the Canadian Institutes for Health Research provided within the survey, it raised the question of whether all respondents considered the study's official definition of “inclusive language” in place of their personal definitions. Though most participants indicated that they used inclusive language prior to their anatomy course, they were not asked to clarify what that meant to them, which prevented the authors from determining if these definitions were aligned. Future work should qualitatively explore how individuals' varied definitions of inclusive terminology impact their reported use of inclusive language, and additionally, assess learners' inclusive language behavior through objective means.

Aside from continuing to use sex and gender primers in anatomy courses, other strategies could be developed to better engage students in learning about diversity as well as practicing using inclusive language. Results of this study indicated that peer interactions and formal workplace/educational training were the most prevalent sources of prior learning about gender and sex diversity. Because these methods are already familiar to most participants, applying them within anatomical education may help learners translate their previously gained knowledge of inclusivity and diversity into an anatomical context. Encouragingly, peer learning has specifically been explored in the literature. A systematic review in the context of practical healthcare education revealed that peer learning helped students to develop their confidence and teamwork capabilities, especially when supplemented with expert‐led learning from clinicians or professors.^21^ Considering that most respondents believed their professors consistently modeled inclusive language and/or represented sex and gender diversity in their courses, they may perceive anatomy educators as ‘experts’ in inclusive anatomical language who could thereby support peer‐learning endeavors. Furthermore, peer‐assisted learning encourages students to actively engage with the content and develop an interest in it, specifically if they are acting as peer tutors.^22^ Using peer learning alongside instructor‐facilitated discussions could thus engage students who felt that their learning experience was unchanged by the addition of diversity content, as well as those who reported no change in their knowledge of sex and gender diversity or inclusive language. Peer‐learning strategies or peer role‐play could also provide learners with a safe setting to practice inclusive language skills, helping to reduce the fear of ‘saying the wrong thing’ which often deters participation.^13^ However, given that more than half of participants felt their peers did not consistently use inclusive language, the value of peer learning may be undermined due to a perceived lack of effort within the group. Pertinently, this might include faculty. Though most educators were perceived to adequately model inclusive language, some reverted to binary terminology or never modeled inclusive language at all despite providing a gender and sex primer to learners.

University educators may face additional difficulties when implementing inclusive language given that academics are chronically overworked.^23^ Retiring binary, exclusionary language requires vigilance, even among those committed to language change.^24^, ^25^ Additionally, a recent review argued that inclusive language is harder to adopt when it is complex, especially when one is scrutinized by an audience.^26^ Thus, exhausted educators may view the provision of a gender and sex primer without modeling inclusive language as the most accessible option, allowing them to incorporate diversity while reducing cognitive load and risk for error. However, students' perceptions of peer learning or other inclusivity interventions hinge on consistent modeling of inclusive language by “experts,” like instructors. As such, the feasibility of peer‐assisted learning, language modeling, and other familiar methods of diversity education should be explored in a quality improvement approach to determine if they are viable for both students and educators to implement in our current academic climate.

Most participants believed that their course materials consistently represented diversity. However, just under one‐quarter indicated that course materials were inconsistently representative of diverse bodies, and that anatomical texts did not reflect principles of diversity. Such responses are consistent with the literature, which shows that anatomical atlases focus heavily on the Eurocentric ideal of the white, lean, body assigned male at birth,^10^, ^16^, ^27^ contributing to a lack of diverse imagery to visualize or describe the human body.^10^ Learners develop their perceptions of “normal” anatomy based on what they see in their textbooks and lectures, or during anatomical dissection,^10^ and this is informed by the homogeneity of bodies that they are exposed to.^28^ So, if the provided learning resources do not align with the instructor's introduction to sex and gender diversity, which would highlight the potentially spectral nature of anatomy, students may perceive inconsistencies in their course materials. Encouragingly, initiatives like POP‐AART,^29^ Western's Health Education Media Library (https://ir.lib.uwo.ca/healthedu), and the Inclusive Anatomy diverse image repository (https://www.inclusiveanatomy.com/image‐repository) will offer accessible, inclusive anatomical imagery, providing educators with more resources to represent diverse bodies in their courses. Future work should assess students' perceptions of inclusive course materials following the incorporation of these resources into anatomy curricula. We also recommend that anatomy educators audit their course materials to ensure that images and language align with their inclusive messaging, to more consistently model these values to students.

### Limitations

This study is limited by its small sample size. Despite recruiting from a pool of several hundred anatomy students across two faculties and multiple levels of study, only 73 survey responses were received. Additionally, the sample was relatively homogenous, predominantly composed of cisgender women, and no sex‐ or gender‐diverse students participated. As such, the perspectives of the population of interest were not captured, and findings may reflect a limited range of experiences. Though a range of responses was observed, the sensitive nature of the topic may have contributed to self‐selection bias, whereby individuals with more polarized views were more likely to participate.

As this study relied on self‐reported data, responses reflect participants' perceptions rather than their objective knowledge or behaviors. While steps were taken to support content and response process validity, the absence of formal psychometric validation may influence how consistently participants interpreted survey items. As such, findings should be interpreted as descriptive and exploratory rather than generalizable.

Additionally, although published definitions of sex and gender were provided within the survey, participants' beliefs may have influenced their reported use of inclusive language. Similarly, the use of sex and gender diversity as an umbrella construct may obscure differences in how participants interpret sex and gender, limiting the specificity of these findings.

### Future Directions

This study provides one of the first exploratory assessments of students' perceptions of sex and gender diversity content in anatomical education. To address current limitations, future work could assess not only students' self‐perceptions of knowledge and language use, but also objectively measure these outcomes to determine whether diversity primers improve learners' understanding. Additionally, qualitative methods should be employed to generate more nuanced insights into participants' definitions, motivations, and behaviors related to inclusive language use. Interviews, in particular, would enable deeper exploration of how learners believe sex and gender diversity content should be incorporated into anatomy curricula, supporting a quality improvement approach to refine existing tools such as diversity primers and peer‐assisted learning strategies. Focus groups may further support this work by facilitating dialogue among learners whose first exposure to diversity concepts occurred during their anatomy course.

Future studies should also consider examining sex diversity and gender diversity separately to capture potentially divergent perspectives. Collectively, these approaches would support more intentional and effective integration of sex and gender diversity into anatomy education, moving beyond supplemental inclusion toward embedded curricular design. Such integration may include early introduction of terminology, consistent role modeling of inclusive language across course materials, and the use of case‐based learning that reflects diverse bodies and identities.

## CONCLUSIONS

In conclusion, our findings indicate that incorporating sex and gender diversity into anatomy courses is feasible and generally beneficial for learners' awareness of equity and inclusivity. Most learners appreciated or were receptive to these efforts, and many reported increased understanding and intent to use inclusive language. Integrating sex and gender diversity content into anatomical education through other methods, such as peer learning, could help to address the practical concerns presented in healthcare professions education literature. Though it may be challenging in the current academic climate, we recommend that anatomy educators make an effort to holistically integrate sex and gender diversity content and model inclusive language for students, as doing so could facilitate peer‐assisted learning, foster a more welcoming learning environment, and better prepare future health professionals to provide equitable care to their diverse patient populations.

## AUTHOR CONTRIBUTIONS

**Charlie W. Brake:** Conceptualization; methodology; writing – original draft; writing – review and editing; investigation; data curation; visualization; formal analysis. **Charys M. Martin:** Conceptualization; methodology; writing – review and editing; supervision; resources; investigation; funding acquisition.

## CONFLICT OF INTEREST STATEMENT

The authors have no conflicts of interest to disclose.

## ETHICS STATEMENT

This study was approved by Western University's Non‐Medical Research Ethics Board (Study #124162).

## Data Availability

Research data are not shared.
